# Genome-Wide Identification and Evolutionary Analysis of Sucrose Synthase (*SUS*) Gene Family in U’s Triangle *Brassica* Species

**DOI:** 10.3390/plants15081224

**Published:** 2026-04-16

**Authors:** Li Huang, Jing-Hui Zhao, Ting Xian, He-Yun Ye, Yu-Fei Xue, You-Rong Chai

**Affiliations:** 1 Integrative Science Center of Germplasm Creation in Western China (Chongqing) Science City and Southwest University/College of Agronomy and Biotechnology, Southwest University, Chongqing 400715, China; orcinus1@163.com (L.H.); jinghuizhao0306@163.com (J.-H.Z.); xiantxxc@163.com (T.X.); 13216270069@163.com (H.-Y.Y.); 2 Engineering Research Center of South Upland Agriculture, Ministry of Education/Academy of Agricultural Sciences, Southwest University, Chongqing 400715, China; 3 Integrative Science Center of Germplasm Creation in Western China (CHONGQING) Science City, Chongqing Technology Innovation Center of Breeding, Southwest University, Chongqing 400715, China

**Keywords:** abiotic stresses, *Brassica*, expression analysis, gene family, phytohormones, sucrose metabolism, sucrose synthase (SUS)

## Abstract

The sucrose synthase (*SUS*) gene family plays a pivotal role in plant carbon metabolism, growth, and development. In this study, we identified 65 *SUS* genes across six *Brassica* species (*B. rapa*, *B. nigra*, *B. oleracea*, *B. juncea*, *B. napus*, and *B. carinata*), and systematically analyzed their structural characteristics, evolutionary history, and expression profiles. Phylogenetic analysis classified these genes into three subfamilies (*SUS*I, *SUS*II, and *SUS*III). *SUS4* orthologs (from *SUS*I subfamily) are completely lost in *Brassica*, and total *SUS* gene numbers are just 6–7 in *Brassica* diploid species, though the *SUS*III subfamily exhibits significant expansion in *Brassica* polyploid species. Selection pressure analysis (Ka/Ks) revealed that the *Brassica SUS* family has primarily undergone purifying selection, although certain members show evidence of adaptive evolution. Comprehensive expression profiling and qRT-PCR validation demonstrated the functional diversification of *BnSUS* genes in tissue specificity and responses to hormonal and abiotic stimuli. *SUS*I genes *BnSUS1-1/2/3/4* are predominantly expressed in vegetative tissues and flowers; *SUS*II genes *BnSUS2-1/2* and *BnSUS3-1/2* are reproductive-organ-specific, while *SUS*III genes *BnSUS5-1/2* and *BnSUS6-1/2/3/4* show young-plant-specific weak expression. *BnSUS* family genes are generally upregulated by ABA, TZ and GA but downregulated by IAA, ACC, BL and JA. Salt, drought, freezing and cold mainly upregulate the *BnSUS* family, heat downregulates it, and osmotic stress exerts both effects. Correspondingly, *Brassica SUS* promoters are enriched with light-responsive (G-box, Box-4), hormone-responsive (ABRE, CGTCA-motif) and anaerobic-induction (ARE) elements. Functional characterization demonstrated that the ABA-responsive gene *BnSUS3-2* significantly improved tolerance to osmotic and ionic stresses by promoting root growth in transgenic *A. thaliana* seedlings. These findings underscore the essential roles of *BnSUS* genes in maintaining cellular homeostasis and provide a theoretical foundation for the genetic improvement of carbon metabolism and stress resilience in *Brassica* crops.

## 1. Introduction

As the main product of plant photosynthesis, sucrose plays a crucial role in plant growth and development. It is the main form of carbohydrate transported from source organs (mainly leaves) to sink/reservoir organs (such as seeds and fruits) [1]. Sucrose not only provides energy and carbon skeletons for plant growth, but also participates in the regulation of various physiological processes [2]. Its decomposition products are involved in (1) the biosynthesis of biological macromolecules such as cellulose, starch and proteins, (2) participation in signal transduction by regulating the expression of microRNAs, transcription factors and related genes, and (3) synergistic working with pathways such as hormone regulation, oxidative stress and defense responses. In agricultural applications, the biosynthesis and decomposition pathways of sucrose directly affect the production efficiency of food, fiber, feed and bioenergy, which is very important for modern agriculture and sustainable development. Studies have shown that approximately 90% of the biomass in plants comes from carbohydrates produced by sucrose metabolism, making it a key determinant of crop yield formation [3]. Therefore, in-depth research on sucrose metabolism holds theoretical and practical significance for enhancing agricultural production efficiency, improving food processing techniques, and developing new types of bioenergy.

The catabolism of sucrose in plants is mainly catalyzed by two enzymes: sucrose-converting enzyme (INV) and sucrose synthase (SUS). SUS or INV can degrade sucrose transported to sink tissue cells into monosaccharides, providing substrates for cell metabolism, biosynthesis, storage and signal transduction [4]. While the hydrolysis of sucrose into glucose and fructose catalyzed by INV is irreversible and directly provides hexoses for cellular metabolism, SUS mediates the reversible reaction sucrose + UDP ↔ UDP-glucose + fructose. As a glycosyltransferase (GT), SUS can not only promote the biosynthesis of sucrose but also decompose sucrose when the carbon demand is high. Its catalytic products are used for energy metabolism and the biosynthesis of primary metabolites, polysaccharides and various secondary metabolites [2,5].

The reversible reaction catalyzed by SUS is crucial in the regulation of carbon allocation. Studies have shown that a decrease in SUS activity can lead to slow plant growth, reduced starch/cellulose synthesis, decreased anaerobic tolerance, and abnormal leaf morphology. Its overexpression can promote plant growth, increase xylem thickness, and enhance cellulose and starch content [2]. For example, in carrots (*Daucus carota* L.), both the leaves and roots of the plants became significantly smaller after reducing the activity of SUS [6]. In cotton (*Gossypium hirsutum* L.), the sucrose synthase gene regulates carbohydrate allocation and ovular development, and is the rate-limiting factor for fiber initiation and elongation. Downregulation of its expression can lead to impaired fiber initiation, elongation and seed development [7,8]. In the hybrid poplar (*Populus trichocarpa* × *Populus deltoides*), the overexpression of cotton *SUS* significantly enhanced the SUS activity in the xylem, increasing the cellulose content of the secondary wall by 2–6% without affecting the plant growth [9]. These findings not only clarify the important role of SUS in the growth and development of plants, but also indicate that SUS has significant potential in the improvement of agricultural traits.

The *SUS* gene family of plants is usually small, but there are significant differences in the number of members among different species. This difference reflects the dynamic changes in the *SUS* gene family during the evolutionary process. The model plants *Arabidopsis thaliana* and rice (*Oryza sativa*) each contain six *SUS* genes, and these genes can be classified into different subfamilies based on their sequence characteristics and expression patterns [10,11]. The *SUS* gene family can be divided into three evolutionary clades, which are widely present in both monocot and dicot plants [2]. In tomato (*Solanum lycopersicum*), rubber tree (*Hevea brasiliensis*), pineapple (*Ananas comosus* (L.) Merr.), kiwifruit (*Actinidia chinensis* and *A. eriantha*) and cacao (*Theobroma cacao* L.), six *SUS* genes have also been identified in their genomes [12,13,14,15,16]. The *SUS* gene family in other species also shows rich diversity. For example, the genome of sweet potato (*Ipomoea batatas*) contains nine *SUS* genes, and the genome of cassava (*Manihot esculenta* Crantz) contains seven *SUS* genes, while the pomegranate (*Punica granatum* L.) and the lychee (*Litchi chinensis*) genomes have only five *SUS* members [17,18,19,20]. This quantitative difference may reflect the different demands in different species for the regulation of sucrose metabolism during evolution, and may also be closely related to the unique genomic evolutionary history of each species, such as polyploidization events and gene loss. It is notable that the number of *SUS* genes often shows a significant increasing trend in polyploid plants. For example, both diploid cotton species *G. arboreum* L. and *G. raimondii* Ulbr. contain eight *SUS* members, but the heterotetraploid cotton species *G. hirsutum* contains as many as 15 *SUS* genes [21]. Among other polyploid plants, poplar (*Populus*) also contains 15 *SUS* members, while tobacco (*Nicotiana tabacum*) and mustard (*Brassica juncea*) contain 14 *SUS* genes [22,23,24]. This expansion in the number of *SUS* genes is very likely related to genome-wide duplication events during the process of species evolution.

The functional research of the *SUS* gene family in plants is relatively in-depth, but there is still a gap in the systematic analysis of the *SUS* gene family in *Brassica* species. Brassicaceae is an important group of plants, including the model plant *A. thaliana*. The genus *Brassica* of this family contains three diploid basal species—*B. rapa* (AA, 2n = 20), *B. nigra* (BB, 2n = 16), and *B. oleracea* (CC, 2n = 18)—as well as three allotetraploid species formed by their inter-species hybridization: *B. juncea* (AABB, 2n = 36), *B. napus* (AACC, 2n = 38) and *B. carinata* (BBCC, 2n = 34) [25]. According to the U Triangle model [26], the three tetraploid species were formed by hybridizations between diploid ancestors (genomes A, B and C). Although different species share the A, B, and C genomes, there are significant genetic variations among them. Therefore, researchers have proposed the concept of “subgenomes” and distinguished homologous genomes in different species through species first-letter superscripts (such as A^r^, A^n^, etc.) [25,27,28].

*Brassica* plants are not only important food, vegetable and ornamental crops, but also have the potential to be used as raw materials for biofuels, playing a key role in human diet, oil production, sightseeing and biofuel development [29]. As one group of the earliest domesticated crops, *Brassica* plants have had a profound influence in agricultural history. The frequent replication and rearrangement of their genomes make them an ideal system for studying the evolution of plant genomes [30]. *B. juncea* has been cultivated in Asia for thousands of years [28]. It has excellent characteristics such as strong stress resistance and low pod breakage rate, especially excelling in adapting to climate changes such as high temperatures. Therefore, it has received increasing attention worldwide. Although *B. napus* has only a few hundred years of domestication history, it has rapidly become the dominant oil crop worldwide due to its high-yield and high-quality seeds, and plays an important role in biodiesel production [31]. In addition, *B. carinata* is often used for germplasm improvement of crops such as *B. rapa*, *B. juncea* and *B. napus* due to its excellent stress resistances [27,29]. It also has phytoremediation properties and is suitable for environmental improvement in marginal and polluted areas [32].

In this study, we identified 65 *SUS* genes in six *Brassica* species on a genome-wide scale and analyzed their properties, structures, evolution rules and expression patterns. It lays a foundation for further studies on the potential functions of *Brassica SUS* genes in various physiological processes, which is important for crop improvement.

## 2. Results

### 2.1. Identification and Basic Properties of SUS Gene Families from Brassica

A total of 65 *SUS* genes were identified in six *Brassica* species, and their quantity distribution showed a significant correlation with genomic ploidy of the species. Among diploid species, *B. rapa*, *B. nigra* and *B. oleracea* contain seven, six, and seven *SUS* genes, respectively. The tetraploid species, *B. juncea*, *B. napus* and *B. carinata*, contain 14, 14 and 17 *SUS* genes respectively, showing a significant increasing trend when compared with diploid species. It is worth noting that the total number of *SUS* genes in tetraploid species is basically consistent with the sum of those from two diploid ancestors, indicating that the *SUS* gene family has been relatively completely preserved after polyploidization. These genes were systematically named based on their homology with the *SUS* genes of *A. thaliana* and their chromosomal positions, and the basic characteristics of the corresponding proteins were analyzed (Table 1). The results show that the protein lengths encoded by these genes range from 476 to 1363 amino acids, and the theoretical isoelectric points (*pI*) range from 5.67 to 8.45. These characteristics are highly consistent with the properties of SUS family proteins reported in other species.

### 2.2. Phylogenetic Relationship Analysis of Brassica SUS Genes

**Figure 1:**
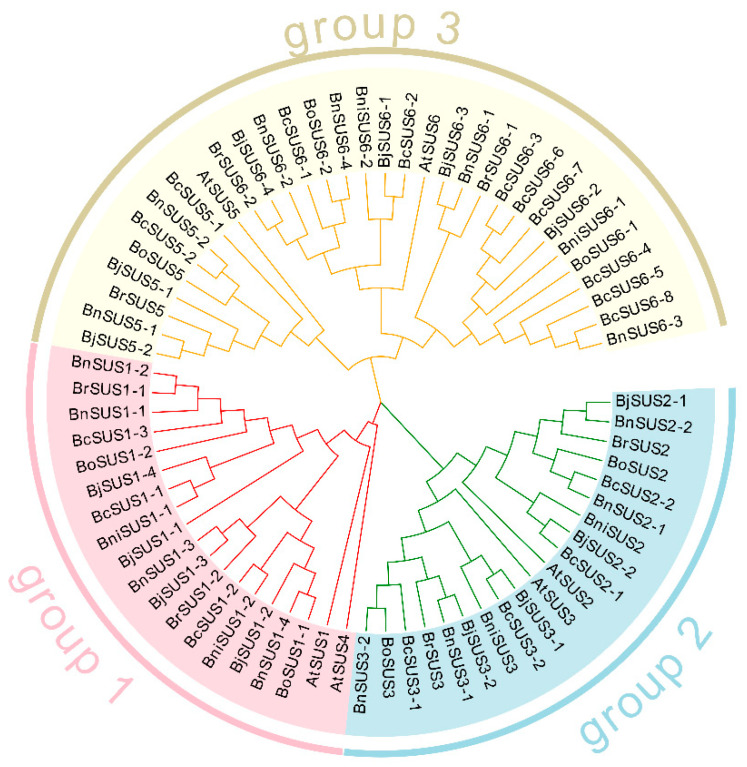
Phylogenetic analysis of SUS family proteins from *Arabidopsis* and six *Brassica* species. Different branch colors denote distinct evolutionary groups: group 1 (SUSI, pink), group 2 (SUSII, blue), and group 3 (SUSIII, yellow).

A phylogenetic tree of SUS proteins from *A. thaliana* and the six *Brassica* species was constructed to determine the evolutionary relationship of this gene family. As shown in Figure 1, all SUS family proteins can be clearly divided into three main evolutionary branches: group 1 (SUSI), group 2 (SUSII) and group 3 (SUSIII). The SUS family of *A. thaliana* and *B. nigra* contains two members in each group, showing a relatively balanced distribution pattern. The SUS family distribution characteristics of *B. rapa* and *B. oleracea* are similar: there are two members in each of group 1 and group 2, and three members in group 3. The SUS family of the tetraploid species *B. juncea* and *B. napus* contains four members each in group 1 and group 2, and six members in group 3. The distribution of SUS family members in *B. carinata* is rather unique: there are 3, 4 and 10 members in group 1, group 2 and group 3, respectively. It is worth noting that *SUS4* orthologs (from *SUS*I subfamily) have been completely lost in *Brassica*.

The number of *SUS* genes in group 3 was significantly higher in all the seven species than in the other two groups. This phenomenon suggests that this subfamily may have experienced significant genetic expansion events during the evolution of *Brassica* species, which may be closely related to functional differentiation or environmental adaptive evolution. The number of *SUS* genes in tetraploid species is basically consistent with the sum of the numbers of genes in their diploid ancestors. This discovery provides strong evidence for genomic conservation during polyploidization. Furthermore, homologous genes of different species tend to cluster in the same evolutionary branch, indicating that these genes have a high degree of conservation during the evolutionary process.

### 2.3. Structural Conservation and Domain Architecture of the Brassica SUS Genes and Proteins

**Figure 2:**
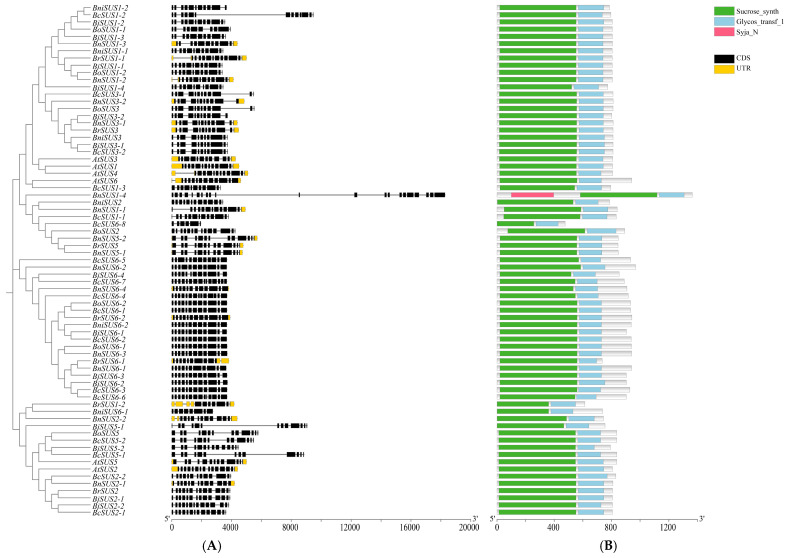
Gene structure and protein domain architectures of the *SUS* gene family from *A. thaliana* and six *Brassica* species. (**A**) Structural organization of *SUS* genes. Exons, introns, and predicted untranslated regions (UTRs) are represented by black boxes, solid lines, and orange boxes, respectively. (**B**) Predicted functional domains of SUS proteins. The green, blue, and pink rectangles denote the Sucrose_synth domain, Glycos_transf domain, and Syja_N domain, respectively.

The structural organization and protein domains of the *SUS* gene family were systematically characterized to understand their functional conservation (Figure 2). Gene structure analysis revealed that most *SUS* members across the seven species exhibit a highly conserved exon–intron pattern, typically containing 10–15 CDS fragments. Specifically, *A. thaliana* (12–15), *B. oleracea* (10–14), *B. juncea* (11–14), *B. rapa* (10–14), *B. nigra* (11–13), *B. napus* (11–13), and *B. carinata* (11–15) showed consistent structural frameworks. However, significant structural variations were identified in certain members, such as *BcSUS6-8* (six fragments) and *BnSUS1-4* (21 fragments), suggesting that these genes may have undergone exon rearrangement or alternative splicing events during gene-specific evolution.

At the protein level, 12 conserved motifs were identified, with the majority of SUS members (63/71) harboring the complete set. Domain architecture analysis confirmed that 70 out of the 71 members contain the canonical Sucrose_synth and Glycos_transf domains, which are essential for sucrose metabolism. Notably, BnSUS1-4 represents a unique evolutionary variant. It is not only the longest sequence in the family, but also contains a unique Syja_N domain at its N-terminus, a feature absent in all other members. These findings indicate that while the core metabolic function of the *Brassica* SUS family is strictly conserved, specific homologs have developed structural complexities that may lead to neo-functionalization.

### 2.4. Promoter Cis-Acting Elements of Brassica SUS Genes

**Figure 3:**
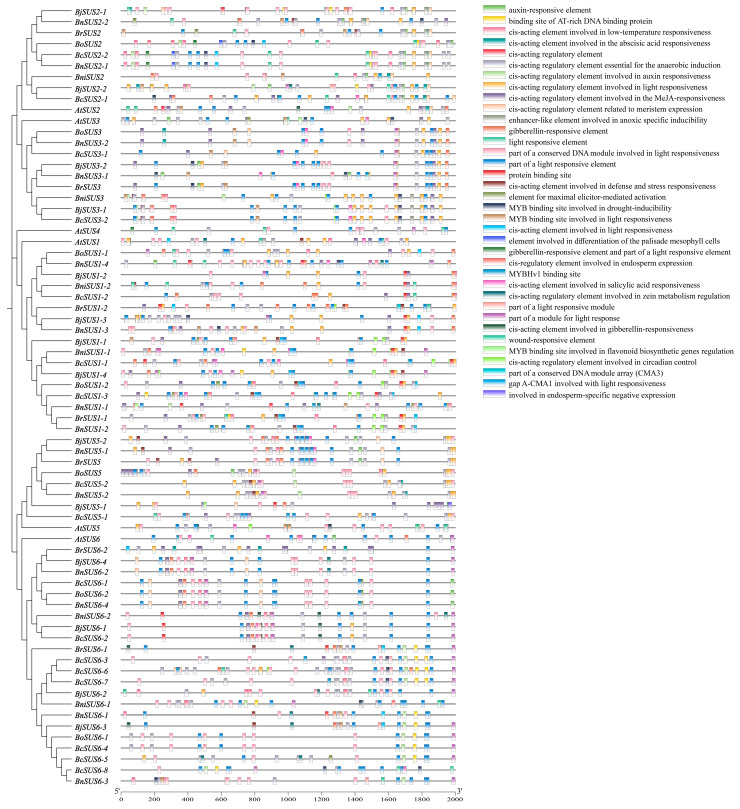
Distribution and functional classification of *cis*-acting regulatory elements in the promoter regions of *SUS* genes across six *Brassica* species and *A. thaliana*. The diagram illustrates the spatial arrangement and diversity of various *cis*-acting elements identified within the 2 kb upstream regions from the translation start site (TSS) of the *SUS* gene family.

To elucidate the transcriptional regulatory mechanisms of *SUS* genes across the seven studied species, we identified and characterized the *cis*-acting elements within the 2000 bp upstream promoter regions using the PlantCARE database. These elements were functionally categorized into five groups: light-responsive (24 types), phytohormone-responsive (nine types), stress-responsive (six types), development-related (six types), and other functional elements (eight types) (Figure 3). Notably, light-responsive elements exhibited the highest abundance, occurring 881 times, with core motifs such as Box-4 (208), G-box (196), GATA-motif (117), and TCT-motif (93) being prominently represented (Figure S1). This enrichment underscores the fundamental role of optical signals in modulating the transcriptional expression of *SUS* genes and perhaps the sucrose synthase activity.

Beyond light regulation, the *SUS* promoters harbor a diverse array of motifs that integrate hormonal and environmental signals. The widespread presence of the abscisic acid-responsive element (ABRE, 181 occurrences), alongside MeJA-responsive elements (CGTCA and TGACG motifs, 92 occurrences each), provides a molecular framework for the observed sensitivity of *BnSUS* members to phytohormone treatments. For instance, the high frequency of ABREs aligns with the strong transcriptional response of *BnSUS3-1/2* to ABA and drought stress. Furthermore, the anaerobic-induction element (ARE) was identified as the most frequent non-light element (211 occurrences), indicating a critical role for the *SUS* family in metabolic adaptation to hypoxia. Collectively, this complex regulatory network suggests that *SUS* genes serve as vital integration points between plant development, hormonal signaling, and environmental adaptation in *Brassica* species.

### 2.5. Chromosome Localization of Brassica SUS Genes

As illustrated in Figure S2, the chromosomal distribution of *SUS* genes of the seven investigated species reveals distinct patterns of genomic organization. In the model plant *A. thaliana*, the six *AtSUS* members are dispersed across four linkage groups (LGs). Among the diploid *Brassica* species, significant divergence in gene distribution was observed. The seven *BrSUS* genes are scattered across six LGs in *B. rapa*, whereas the six *BniSUS* genes in *B. nigra* are concentrated within only three LGs. In *B. oleracea*, seven *BoSUS* genes are distributed across four LGs, with a notable localized gene cluster identified on chromosome C06, which harbors three homologs. These findings underscore the prominent interspecific variation in *SUS* gene distribution even among closely related diploid ancestors.

The transition from diploidy to allopolyploidy is accompanied by a marked expansion in both the number and genomic range of *SUS* genes. In the tetraploid species, 14 *BjSUS* genes (in *B. juncea*) and 14 *BnSUS* genes (in *B. napus*) are distributed across 10 LGs each. In *B. carinata*, 17 *BcSUS* genes are mapped to seven LGs and one scaffold. This expanded distribution pattern likely reflects extensive genomic recombination and gene duplication events associated with polyploidization. Notably, the presence of several *SUS* homologs on unanchored genomic scaffolds suggests either ongoing challenges in genome assembly or the existence of complex and species-specific chromosomal structural variations.

### 2.6. Genomic Collinearity and Evolutionary Analysis of Brassica SUS Genes

**Figure 4:**
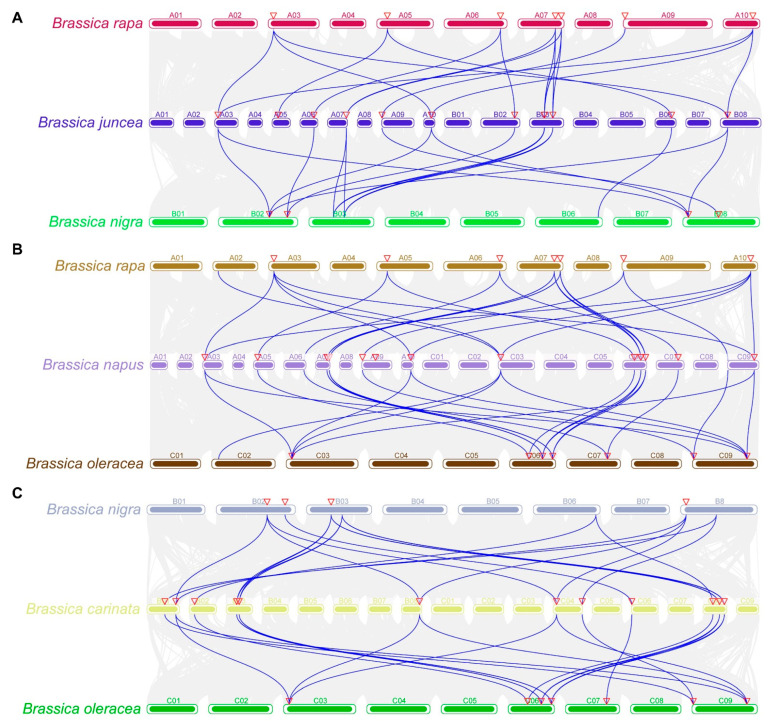
Synteny analysis of the *SUS* gene family among multiple *Brassica* genomes. Collinear relationships were identified across three distinct genomic combinations: (**A**) *B. rapa* (AA), *B. juncea* (AABB), and *B. nigra* (BB); (**B**) *B. rapa* (AA), *B. napus* (AACC), and *B. oleracea* (CC); and (**C**) *B. nigra* (BB), *B. carinata* (BBCC), and *B. oleracea* (CC). The arc-shaped blocks represent the chromosomes of each species, while the background gray curves denote the global syntenic blocks between genomes. Blue lines specifically highlight the syntenic gene pairs associated with the *SUS* gene family, illustrating their evolutionary conservation and retention patterns during polyploidization.

To explore the evolutionary mechanism of the *SUS* gene family in *Brassica* species, we systematically compared the distribution of *SUS* genes between diploid ancestral species and their derived polyploid species through genome-wide collinearity analysis (Figure 4). Specifically, it includes the following three groups of analyses: (1) *B. rapa* (AA), *B. juncea* (AABB), and *B. nigra* (BB); (2) *B. rapa* (AA), *B. napus* (AACC) and *B. oleracea* (CC); (3) *B. nigra* (BB), *B. carinata* (BBCC) and *B. oleracea* (CC).

Our analysis shows that the *SUS* genes exhibit a specific retention pattern in polyploid species. In the AA-AABB-BB group, there were 18 pairs of collinear genes between AA and AABB, while there were 16 pairs between BB and AABB. This indicates that during the formation of AABB, the subgenome from AA may have a higher gene retention rate. The *SUS* genes of the AA subgenome may be more functionally important or be subjected to greater selection pressure. In the AA-AACC-CC group, the number of collinear gene pairs between AA and AACC was 15, while it was as high as 19 pairs between CC and AACC. This indicates that the CC subgenome may have experienced stronger selection pressure during the evolution of AACC, resulting in more complete preservation of the *SUS* genes. Furthermore, in the BB-BBCC-CC group, there were only 15 pairs of collinear genes between BB and BBCC, while there were 20 pairs between CC and BBCC, further supporting that the CC subgenome has a higher gene retention rate after polyploidization.

### 2.7. Selection Pressure of Brassica SUS Genes

To explore the evolutionary selection pattern of the *SUS* gene family in the U Triangle species, we analyzed the selection pressure among different genomes by calculating the ratio of non-synonymous replacement rate (Ka) to synonymous replacement rate (Ks) of homologous gene pairs (Ka/Ks). Results are shown in Table S1; the Ka/Ks values of all homologous gene pairs were significantly less than 1 (AA-AABB mean 0.1022, BB-AABB mean 0.1280; AA-AACC mean 0.1110, CC-AACC mean 0.1045; BB-BBCC mean 0.1294, and CC-BBCC mean 0.1418), indicating that the *SUS* gene family has been mainly subjected to purifying selection during evolution, and its protein sequences are highly conserved and their functions may be strictly constrained. It is notable that a few gene pairs (such as *BoSUS6-1*/*BcSUS6-4*, Ka/Ks = 0.4593; *BoSUS1-2*/*BcSUS1-3*, Ka/Ks = 0.4938) showed a relatively high ratio close to 0.5, suggesting that these genes might have undergone relaxed selection or local positive selection, which might be related to the functional differentiation after polyploidization.

Further analysis revealed that there was no significant difference in Ka/Ks values between polyploid species (AABB, AACC, BBCC) and their diploid ancestors (AA, BB, CC), indicating that polyploidization events did not significantly alter the selection pressure of the *SUS* genes. In conclusion, the *SUS* gene family is generally dominated by purifying selection in the species of the U Triangle, but some genes may undergo adaptive evolution after polyploidization, providing clues for subsequent studies on their functional differentiation.

Statistical summary of the Ka/Ks analysis (Table 2) showed that the mean values for all six comparison groups were consistently low, ranging from 0.1015 to 0.1541. Notably, 100% of the 103 identified *SUS* gene pairs exhibited Ka/Ks ratios below 0.5, with a predominant distribution around 0.1. This statistical evidence further supports the conclusion that *SUS* genes are under stringent purifying selection across the U Triangle species.

### 2.8. Tissue-Specific Expression Patterns of B. napus SUS Genes

**Figure 5:**
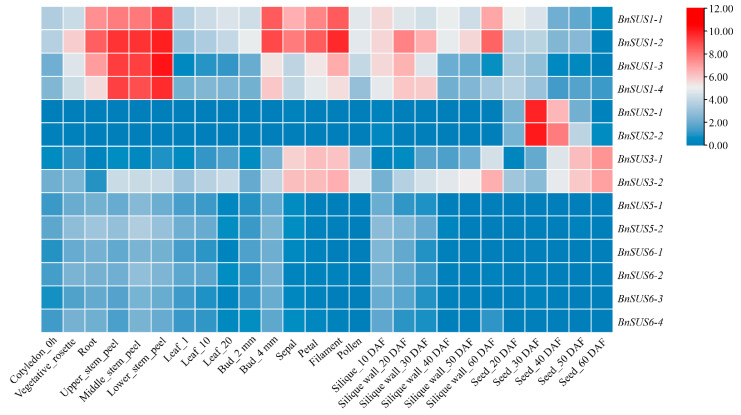
Tissue-specific expression heatmap of *B. napus SUS* genes. Transcriptome data from BnTIR database was used to analyze expression patterns of the *SUS* gene family across different tissues in *B. napus*. Expression levels are shown as log2(TPM + 1) values, with the color gradient ranging from blue (low expression) to red (high expression).

Expression profiling based on the BnTIR database revealed significant functional diversification among *BnSUS* subfamilies across various tissues (Figure 5). The *SUS1* subfamily (*BnSUS1-1*/2/3/*4*) exhibited broad constitutive expression, with particularly high transcript abundance in roots and stems. These members, along with *BnSUS3-1*/*2*, were also prominently expressed in floral organs, including sepals, petals, and filaments, suggesting their involvement in both vegetative growth and reproductive development. *BnSUS3-1*/*2* were the only dominantly expressed *BnSUS* members at the latest stages of developing seeds, and *BnSUS3-2* was expressed in much more diverse tissues than *BnSUS3-1*. In contrast, the *SUS2* subfamily (*BnSUS2-1*/*2*) displayed strict tissue specificity, with significant induction observed exclusively in siliques at 20–50 days after flowering (DAF) with a peak in 30 DAF seeds. Members of the *SUS5* and *SUS6* subfamilies generally maintained low expression levels across all detected tissues, with vegetative organs, young buds and young silique walls being major places for their expression.

**Figure 6:**
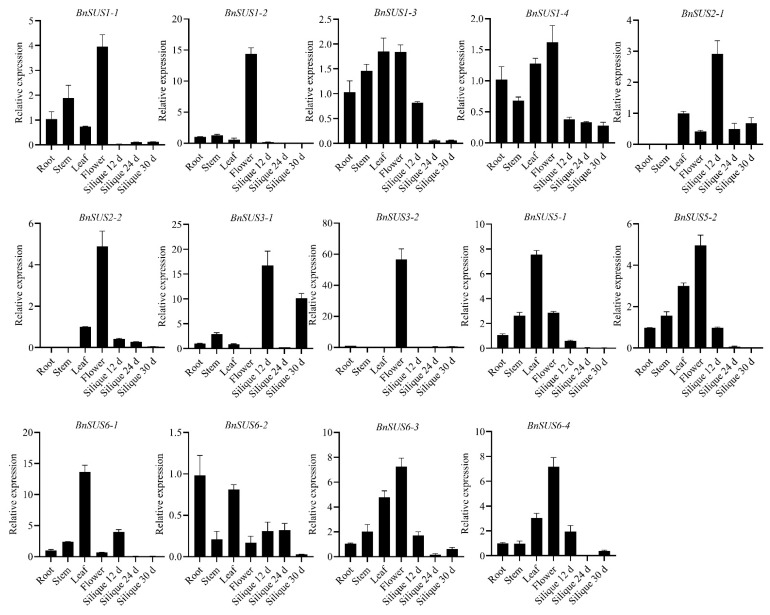
Expression of *BnSUS* genes in different tissues of *B. napus* validated by qRT-PCR. Root, stem, leaf, flower, and siliques at 12, 24, and 30 days after flowering were analyzed.

To validate these bioinformatic predictions, qRT-PCR was performed on representative *BnSUS* genes (Figure 6). The experimental results confirmed that *BnSUS1-1/2, 2-2, 3-2, 5-2, 6-3,* and *6-4* were dominantly expressed in floral tissues, with *BnSUS1-2* and *6-2* showing the highest relative abundance. Notably, the complete absence of *BnSUS2-1*/*2* transcripts in vegetative organs (roots and stems) further confirms the specialized role of this subfamily in reproductive development. Overall, the qRT-PCR data showed high consistency with the database-derived expression patterns, demonstrating the reliability of in silico expression analysis results.

### 2.9. Responsiveness of B. napus SUS Genes to Phytohormones and Abiotic Stresses

**Figure 7:**
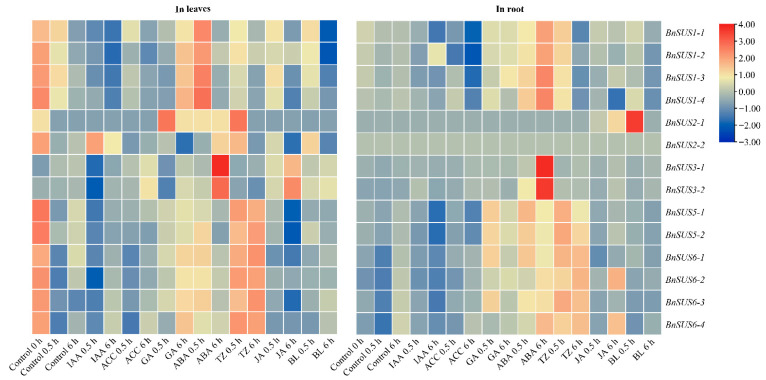
Expression of *BnSUS* genes in leaf and root under various phytohormone treatments. The heat map illustrates the transcriptional responses of the *BnSUS* genes to a diverse range of phytohormones, including IAA (indole-3-acetic acid), ACC (1-aminocyclopropane-1-carboxylic acid), GA (gibberellin), ABA (abscisic acid), TZ (trans-zeatin), JA (jasmonic acid), and BL (brassinolide). Expression levels are represented as log2-transformed TPM (Transcripts Per Million) values, with the color gradient ranging from blue (low expression) to red (high expression).

The transcriptional plasticity of *BnSUS* genes was further evaluated under various phytohormone stimuli (Figure 7). In leaf tissues, all *BnSUS* genes showed drastic variation over time in control (non-treated) conditions, perhaps because of light-responsive circadian rhythm regulation of *SUS* expression [33]. Thus, we could only observe phytohormone-responsive rough trends of *BnSUS* genes in leaves: upregulation by ABA (esp. *BnSUS1-1/2/3/4*, *BnSUS3-1/2* and *BnSUS6-1/2/3/4*), TZ (esp. *BnSUS6-1/2/3/4*, *BnSUS5-1/2* and *BnSUS2-1/2*) and GA (esp. *BnSUS2-1* and *BnSUS6-1/2/3/4*), downregulation by IAA (esp. *BnSUS3-1/2* and *BnSUS1-1/2/3/4*), BL (esp. *BnSUS1-1*/*2*) and JA (esp. *BnSUS5-1/2*), and soft influence by ACC. In roots, expression of *BnSUS* genes (except *BnSUS6-1/2/3/4*) over time in control conditions was relatively stable. In roots, *BnSUS* genes could be upregulated by ABA (esp. *BnSUS3-1/2*, *BnSUS1-1/2/3/4*, *BnSUS5-1/2* and *BnSUS6-1/2/3/4*), TZ (esp. *BnSUS6-1/2/3/4*, *BnSUS5-1/2* and *BnSUS1-1/2/3/4*) and GA (esp. *BnSUS5-1/2* and *BnSUS6-1/2/3/4*), downregulated by ACC (esp. *BnSUS1-1/2/3/4* and *BnSUS5-1/2*) and IAA (esp. *BnSUS5-1/2* and *BnSUS1-1*), and softly influenced by BL (except quick sharp upregulation of *BnSUS2-1*) and JA (except downregulation of *BnSUS1-4* and upregulation of *BnSUS2-1*, *BnSUS6-2* and *BnSUS6-4*). Most of the responsive trends of *BnSUS* family members to phytohormones were similar between leaves and roots, but differences were also distinct.

**Figure 8:**
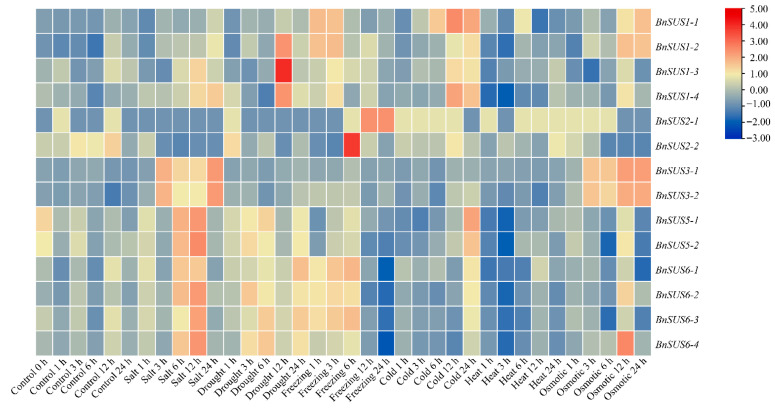
Expression of *BnSUS* genes in *B. napus* leaves under diverse abiotic stress conditions. This heat map summarizes the expression patterns of *BnSUS* genes in response to multiple environmental stressors, including salt, drought, freezing, cold, heat, and osmotic stress. The expression abundance is visualized using log2-transformed TPM values.

Transcriptomic analysis underscored the pivotal involvement of the *BnSUS* family in environmental adaptation (Figure 8). Under salt stress, *BnSUS3-1/2* were quickly upregulated with long durations; *BnSUS5-1/2* and *BnSUS6-1/2/3/4* were also largely upregulated; *BnSUS1-1/2/3/4* were moderately upregulated, while *BnSUS2-1/2* were downregulated. Under drought stress, *BnSUS6-1/2/3/4* and *BnSUS5-1/2* were significantly upregulated with long durations, and *BnSUS1-3/2/4/1* were sharply upregulated after 12 h of treatment. Freezing treatment quickly and considerably upregulated *BnSUS6-1/2/3/4* and *BnSUS1-1/2/3/4* and suddenly upregulated *BnSUS2-1/2* to high levels after 12 h of treatment. Cold stress upregulated *BnSUS1-1/2/3/4* gradually to high levels, and 24 h of cold treatment also upregulated *BnSUS5-1/2* and *BnSUS6-1/2/3/4* substantially. Opposite to cold stress, heat treatment significantly downregulated *BnSUS6-1/2/3/4*, *BnSUS5-1/2* and *BnSUS1-1/2/3/4*. Under osmotic stress, *BnSUS* family members responded diversely: it gradually upregulated *BnSUS3-1/2*, *BnSUS1-1/2* and *BnSUS6-4* to high levels, but downregulated *BnSUS2-1/2*, *BnSUS5-1/2* and *BnSUS6-1/3* considerably.

### 2.10. Identification and Functional Validation of Candidate Gene BnSUS3-2

By integrating evolutionary analysis, promoter *cis*-acting element characterization, and transcriptomic profiling, *BnSUS3-2* was identified as a primary candidate for mediating abiotic stress resilience. The selection of *BnSUS3-2* was predicated on its rapid transcriptional response to drought and salt stress, alongside its pronounced sensitivity to abscisic acid (ABA), which is highly consistent with the enrichment of ABA-responsive elements (ABREs) identified within its promoter region.

Subsequently, *BnSUS3-2* was overexpressed in *A. thaliana* to further validate its function in stress response. Positive transgenic lines were successfully established and verified through genomic DNA PCR and quantitative real-time PCR (qRT-PCR) analysis (Figure S3).

**Figure 9:**
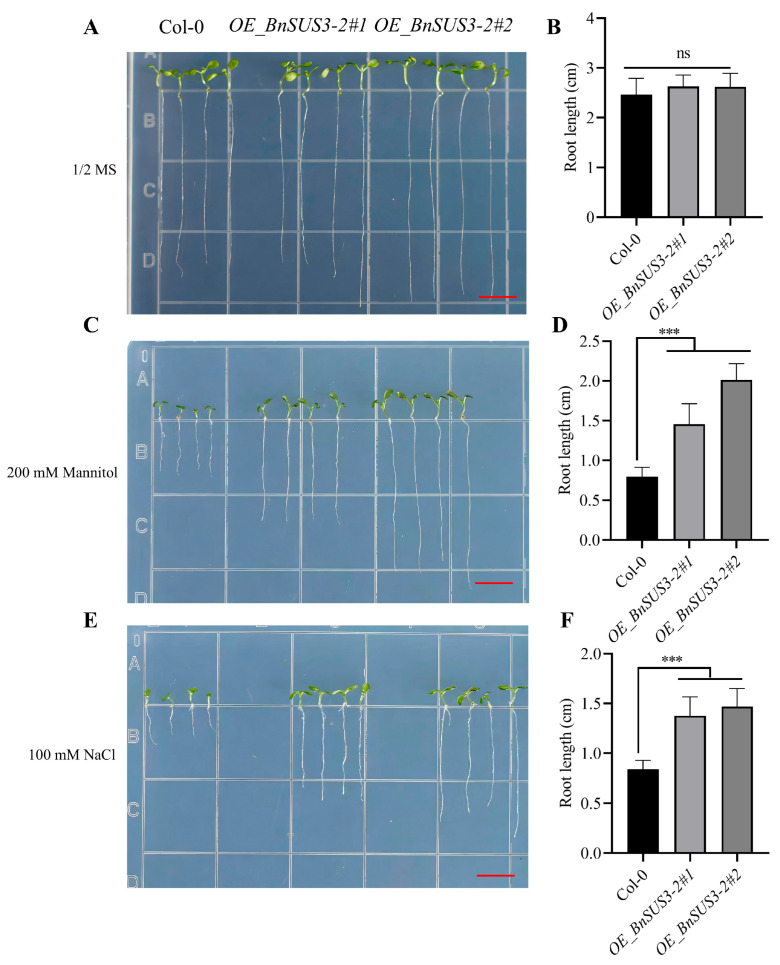
Root growth analysis of transgenic *A. thaliana* under mannitol-induced osmotic stress and salt stress. (**A**,**B**) Root growth phenotype and statistical analysis of root length under control conditions (MS medium). (**C**,**D**) Root growth phenotype and statistical analysis under osmotic stress induced by 200 mM mannitol. (**E**,**F**) Root growth phenotype and statistical analysis under salt stress induced by 100 mM NaCl. All phenotypic observations and measurements were performed on 10-day-old seedlings (n > 20 per treatment). Root lengths were quantified using ImageJ software. (v1.8.0) Error bars represent the standard error, and asterisks (***) indicate a statistically extremely significant difference at *p* < 0.001, and ns indicates no significant difference compared to the wild type (WT) based on Student’s *t*-test. Scale bar = 1 cm.

Phenotypic evaluation of T_3_ homozygous lines revealed that under normal growth conditions (MS medium), the root length of the overexpressing lines was slightly greater than that of the WT, although this difference was not statistically significant (Figure 9A,B). However, under osmotic stress conditions simulated by 200 mM mannitol, the transgenic lines exhibited a significant growth advantage, with root systems being markedly longer than those of the WT (Figure 9C,D). Similarly, when subjected to salt stress induced by 100 mM NaCl, the *BnSUS3-2-*overexpressing lines consistently maintained superior growth vigor, especially root elongation, compared to the control plants (Figure 9E,F). These results substantiate that *BnSUS3-2* functions as a positive regulator of osmotic and ionic stress adaptation, providing a strategic target for the genetic improvement of tolerance to drought and salt in *Brassica* crops.

## 3. Discussion

The *SUS* gene family constitutes a pivotal regulatory hub in plant carbon metabolism, influencing both vegetative growth and reproductive development. In the present study, a comprehensive genome-wide analysis identified 65 *SUS* genes across six *Brassica* species. By integrating evolutionary trajectories, structural divergence, and spatio-temporal expression profiles, we provide a robust theoretical framework for understanding the functional conservation, diversification, neo-functionalization and regulatory complexity of the *SUS* family in polyploid crops.

### 3.1. Expansion of the Brassica SUS Gene Family Driven by Polyploidization

Our results demonstrate a strong positive correlation between *SUS* gene copy numbers and genomic ploidy levels. Diploid species (AA, BB, CC) harbor an average of 6–7 members, whereas allotetraploid species (AABB, AACC, BBCC) possess gene counts that are nearly equal to or exceed the sum of their diploid progenitors (14 in *B. juncea*, 14 in *B. napus*, and 17 in *B. carina*). This pattern aligns with the genomic conservation mechanism typically observed following whole-genome duplication (WGD), suggesting that *SUS* genes are high-retention candidates post polyploidization [34]. Notably, the *SUS* group 3 (*SUS*III) subfamily in *B. carina* (BBCC) exhibits significant expansion (10 members) compared to its ancestors *B. nigra* (BB) and *B. oleracea* (CC) (six members combined). This “local expansion” likely reflects subgenome-specific selection pressures and the asymmetric evolution of the CC subgenome, which may contribute to the unique metabolic adaptability of Ethiopian mustard. On the other hand, orthologs of *AtSUS4* of *SUS* group 1 (*SUS*I) have been completely deleted from all U Triangle *Brassica* species. This means that its function could be replaced by the triplicated *Brassica SUS1* paralogs, or *Brassica* plants could abandon *SUS4* function because of directional evolution. Finally, *SUS* gene numbers in diploid *Brassica* species (seven in *B. rapa*, six in *B. nigra*, and seven in *B. oleracea*) are just a little more than or even equal to the *SUS* gene number (six) in *A. thaliana*. This indicates the deletion of nearly 2/3 of the triplicated *SUS* genes (mainly of *SUS*I and *SUS*II subfamilies) during the long-time rediploidization process of the *Brassica* ancestor after the whole-genome triplication event, and further implies that an *SUS* gene number of 6–7 is enough to support basic functions of a diploid plant species. As for the high *SUS* gene numbers in *Brassica* allotetraploid species, they are the results of recent inter-species hybridizations.

### 3.2. Structural Conservation and Functional Divergence of Brassica SUS Genes

Phylogenetic mapping classified the *Brassica SUS* genes into three distinct groups, consistent with the *Arabidopsis* nomenclature, implying that their fundamental functional differentiation predates the divergence of the *Brassica* lineage. The high conservation of 12 motifs and the presence of quintessential Sucrose_synth and Glycos_transfer domains underscore the stability of their core catalytic roles in sucrose metabolism. However, specific structural innovations were identified: the unique Syja-N domain in BnSUS1-4 and the motif deletions in BcSUS6-8 suggest potential functional recalibration. Furthermore, the 21-exon architecture of *BnSUS1-4* is atypical for this family, providing strong evidence for exon shuffling or rearrangement as a driver for acquiring novel regulatory properties [35].

### 3.3. Diversification of Spatio-Temporal Expressions and Potential Functions of Brassica SUS Genes

The *SUS* gene family in *Brassica* displays pronounced tissue-specific expression, hinting at functional sub-functionalization. Members of the *SUS* group 1 (*SUS*I, e.g., *BnSUS1-1/2/3/4*) are predominantly expressed in vegetative tissues (roots and stems) and flowers, suggesting a primary role in fueling biomass accumulation and initial building of reproductive sink strength. Conversely, *SUS* group 2 (*SUS*II, e.g., *BnSUS2-1/2* and *BnSUS3-1/2*) shows high transcript abundance in reproductive organs, especially *BnSUS2-1/2* in middle-stage developing seeds and *BnSUS3-1/2* in late-stage developing seeds, pointing to a specialized role in seed filling and carbon partitioning. On the other hand, *SUS* group 3 (*SUS*III, e.g., *BnSUS5-1/2* and *BnSUS6-1/2/3/4*) shows only low expression in vegetative organs, young buds and young silique walls.

Most members of the *BnSUS* family respond to various phytohormones and abiotic stresses. When treated with phytohormones, generally speaking, ABA, TZ and GA tend to upregulate, while IAA, ACC, BL and JA tend to downregulate their expression, though a few members are exceptional. When challenged with stresses, most members of the *BnSUS* family could be upregulated by salt, drought, freezing and cold and downregulated by heat, though a few members show opposite trends. Osmotic stress is rather unique, because it could both upregulate and downregulate batches of member genes. This all means that *BnSUS* family genes are key players regulated by phytohormones and environmental stresses to modulate plant development and adaptability. The enrichment of *cis*-regulatory elements in *SUS* promoters further elucidates their regulatory landscape. The prevalence of light-responsive (G-box, Box-4) and hormone-responsive (ABRE, CGTCA-motif) elements suggests that *SUS* expression is finely tuned by environmental and endogenous signals [36]. In particular, the ubiquitous presence of anaerobic-induction elements (ARE) implies a conserved role in hypoxia stress signaling, reinforcing the hypothesis that *SUS* serves as a metabolic bypass to maintain energy homeostasis under abiotic stress.

### 3.4. Purifying Selection and Subgenome Asymmetry of Brassica SUS Genes

The Ka/Ks ratios for almost all homologous pairs ranged from 0.10 to 0.14, indicating that the *SUS* family has been under stringent purifying selection to maintain vital metabolic functions. Nevertheless, the elevated ratio in specific pairs (e.g., *BoSUS6-1/BcSUS6-4*, Ka/Ks = 0.459) suggests localized positive selection or relaxation of functional constraints post polyploidization [2,37]. Collinearity analysis further highlights subgenome dominance; the higher retention rate of *SUS* genes from the CC subgenome in both *B. napus* (AACC) and *B. carina* (BBCC) suggests an evolutionary preference. This subgenomic asymmetry may be governed by functional complementarity or epigenetic buffering, offering a fascinating avenue for future research into polyploid heterosis.

### 3.5. Implications for Crop Improvement and Future Perspectives

The economic productivity of *Brassica* oilseed and vegetable crops is intrinsically linked to the efficiency of sucrose transshipment and utilization. Our systematic analysis of the *SUS* family reveals how polyploidization has fostered gene expansion and functional diversification. The expanded *SUS* group 3 subfamily in *B. carina* represents a prime candidate for enhancing environmental resilience. Our findings specifically highlight *BnSUS3-2* as a primary candidate for mediating abiotic stress resilience, predicated on its rapid transcriptional response to drought and salt [38,39,40]. This hypothesis was rigorously substantiated through its heterologous overexpression in *A. thaliana*, where *OE_BnSUS3-2* lines exhibited a marked growth advantage over WT plants. Under osmotic (200 mM mannitol) and ionic (100 mM NaCl) challenges, the transgenic seedlings maintained significantly more vigorous growth with longer root systems. This phenotype is likely driven by enhanced “sink strength” in the roots; by accelerating sucrose cleavage, heterologous *BnSUS3-2* helps maintain favorable turgor pressure and provides the necessary carbon skeletons for sustained development under water-deficit conditions in transgenic *A. thaliana* seedlings.

Future endeavors should integrate multi-omics approaches to validate the specific contributions of different subfamilies to yield formation by combining transcriptomic and metabolomic flux data. Utilizing CRISPR/Cas9-mediated gene editing or precision overexpression will be essential to decouple the complex regulatory networks of *SUS* genes, ultimately facilitating the development of “high-yield, high-quality and high-resilience” *Brassica* cultivars.

## 4. Materials and Methods

### 4.1. Sequence Retrieval and Identification of SUS Genes from 6 Brassica Species

To identify *SUS* genes, the *A. thaliana* genome TAIR10, the *B. rapa* genome Chiifu_V4.0, the *B. oleracea* genome JZS_V2.0, the *B. juncea* genome tum_V1.5, the *B. nigra* genome CN115125 v1, the *B. napus* genome ZS11.v0 and the *B. carinata* genome zd-1.v0 were downloaded from TAIR database (https://www.arabidopsis.org/, accessed on 24 November 2024), the Brassicaceae Database (http://brassicadb.cn/, accessed on 24 November 2024) and BnIR database (https://yanglab.hzau.edu.cn/, accessed on 24 November 2024). Using the previously published SUS protein sequences of *A. thaliana* as queries [10], the six *Brassica* species databases were extensively searched to identify the candidate *SUS* genes. In addition, BLASTp analysis was done using candidate SUS protein sequences of 6 *Brassica* species to complete further identification.

### 4.2. Phylogenetic Tree Construction

Using MEGA7 software, protein sequences of the *SUS* gene family were aligned and the phylogenetic tree was produced [41]. Finally, the classification of *SUS* genes from *A. thaliana* and these 6 *Brassica* species was performed using iTOL v7 (https://itol.embl.de/, accessed on 30 November 2024)

### 4.3. Analyses of Conserved Motifs and Domains

The conserved motif identification of the SUS proteins was analyzed by MEME suite (http://meme-suite.org/tools/meme, accessed on 12 December 2024) [42], and the conserved domain identification of the SUS proteins was analyzed by SMART (http://smart.embl-heidelberg.de/, accessed on 17 December 2024) [43]. In addition, conserved motifs and domains were visualized using TBtools-II (v2.458) [44].

### 4.4. Analyses of Physical and Chemical Properties of SUS Proteins

In order to analyze the physical and chemical properties of SUS proteins in these seven species, we obtained number of amino acids, molecular weight, theoretical *pI*, instability index, aliphatic index and grand average of hydropathicity using TBtools-II.

### 4.5. Analyses of Gene Structure, Gene Location and Cis-Acting Regulatory Elements

Using TBtools-II based on the GFF3 files of the seven species, gene exon–intron structure and gene location images of *SUS* family genes were generated. The *cis*-acting regulatory elements in the 2000 upstream bases of *SUS* genes were obtained from PlantCARE database (http://bioinformatics.psb.ugent.be/webtools/plantcare/html/, accessed on 4 March 2025), and they were visualized using TBtools-II [45].

### 4.6. Analyses of Collinearity and Evolutionary Constraint Value (Ka/Ks)

Gene pair collinearity was determined using the One Step MCScanX—Super Fast plugin of software TBtools-II. In addition, we used the Multiple Synteny Plot of TBtools-II to generate three collinear images between six *Brassica* species. Finally, using the coding sequence (CDS) files of seven target species and the homologous gene pairs identified through synteny analysis, we calculated the ratio of non-synonymous substitutions (Ka) to synonymous substitutions (Ks) for the homologous gene pairs using the Simple Ka/Ks Calculator (NG) feature module built into TBtools-II.

### 4.7. RNA Extraction and cDNA Synthesis

In this study, *B. napus* cultivar Zhongshuang 11 (ZS11) and *A. thaliana* Col-0 along with *OE_BnSUS3-2* transgenic lines were used as research materials. For *B. napus*, samples were collected from vegetative organs (roots, stems and leaves) and reproductive organs (flowers and siliques at 12, 24 and 30 days after flowering). For *Arabidopsis*, rosette leaves from both Col-0 and *OE_BnSUS3-2* lines were harvested for comparative transcriptional analysis. All collected samples were immediately flash-frozen in liquid nitrogen and stored in an ultra-low-temperature refrigerator at −80 °C for subsequent use. Total RNA was extracted using the RNA simple Total RNA Kit (TIANGEN, Beijing, China) following the manufacturer’s protocol. To eliminate potential genomic DNA contamination, reverse transcription was performed using the PrimeScript™ RT reagent Kit with gDNA Eraser (Perfect Real Time) (TaKaRa, Tokyo, Japan).

### 4.8. qRT-PCR Detection and Gene Expression Level Analysis

Gene expression data were obtained from the BnIR database (https://yanglab.hzau.edu.cn/, accessed on 15 May 2025) and heatmap visualization was performed using TBtool-II software. The qRT-PCR experiment was accomplished using the Bio-Rad CFX Connect Real-Time PCR Detection System. The reaction system followed the ChamQ Blue Universal SYBR qPCR Master Mix reagent from Vazyme Company (Nanjing, China) and three technical replicates were set for each sample. Taking *25SrRNA* as the internal reference gene, the relative expression level of the *BnSUS* genes was calculated by the 2^−ΔΔCT^ method [46]. After the original data were processed by Excel, statistical analysis and bar chart drawing were performed using GraphPad Prism 10.4.2 software. All qRT-PCR primers were designed using the NCBI Primer-Blast tool (https://www.ncbi.nlm.nih.gov/, accessed on 21 May 2025). Primer specificity was verified by melting curve analysis and sequencing. Detailed primer sequences are shown in Table S2.

### 4.9. Vector Construction and Arabidopsis Transformation

The complete coding sequence (CDS) of *BnSUS3-2* was amplified using gene-specific primers (Table S1). The resulting PCR products were subcloned into the pCAMBIA1300 vector, which had been linearized by digestion with *Kpn*I and *Bam*HI, using the ClonExpress^®^ II One Step Cloning Kit (Vazyme, Nanjing, China). The recombinant plasmid, p*35S::BnSUS3-2*, was subsequently introduced into *A. thaliana* Col-0 (wild-type, WT) plants via the *Agrobacterium tumefaciens* (strain GV3101)-mediated floral dip method [47]. Positive transformants were screened on MS medium supplemented with hygromycin (50 mg/L). The progeny of these transformants exhibited an approximate 3:1 segregation ratio of resistant to sensitive phenotypes in the T_2_ generation. Finally, homozygous T_3_ lines were identified and utilized for further physiological and molecular analyses. *Actin8* (*AT1G49240*) was used as the internal reference gene for qRT-PCR in *Arabidopsis*.

## 5. Conclusions

In summary, this study provides a systematic genome-wide identification and evolutionary analysis of the *SUS* gene family within the *Brassica* Triangle of U. A total of 65 *SUS* genes were identified across six *Brassica* species. The *Brassica* diploid species contain only 6–7 *SUS* genes, implying loss of nearly 2/3 of triplicated *SUS* genes during the rediploidization process of *Brassica* ancestor. The *SUS* gene numbers in *Brassica* allotetraploids largely reflect the sum of their diploid ancestors, indicating high genomic conservation during recent polyploidization. Phylogenetic analysis categorized these genes into three distinct subfamilies, among which the *SUS*III subfamily underwent significant expansion in polyploid species. While the majority of members possess the canonical Sucrose_synth and Glycos_transf domains and are under strong purifying selection, the identification of the unique *BnSUS1-4* variant with an N-terminal Syja_N domain and a rare 21-exon structure suggests potential neo-functionalization. Furthermore, the complex landscape of *cis*-acting elements in the promoter regions—dominated by light, hormones (ABA, IAA, MeJA), and anaerobic-induction (ARE) motifs—provides a molecular framework for the observed spatio-temporal and stress-responsive expression patterns. Specifically, the constitutive expression of *SUS1* members in vegetative and flower organs, the strict reproductive organ specificity of the *SUS2* and *SUS3* genes, the young-plant-specific weak expression of *SUS5* and *SUS6* genes, and the diversified broad-spectrum hormonal sensitivity and multiple-stress responsiveness of *BnSUS* family genes highlight the functional diversification of this family in *B. napus*. Subsequently, the heterologous overexpression of the candidate gene *BnSUS3-2* in *A. thaliana* significantly enhanced seedling tolerance to drought and salt stress, as evidenced by markedly increased root elongation under 200 mM mannitol and 100 mM NaCl treatments. This confirms that *BnSUS3-2* functions as a positive regulator of osmotic stress adaptation. Collectively, these findings establish an SUS-related theoretical foundation for the genetic improvement of carbon metabolism and environmental resilience in *Brassica* crops.

## Figures and Tables

**Table 1:** Physicochemical properties of SUS family proteins from *Brassica*.

Name	Locus ID	Number of Amino Acids	Molecular Weight	Theoretical *pI*	Instability Index	Aliphatic Index	Grand Average of Hydropathicity
AtSUS1	*AT5G20830*	808	92,997.62	5.83	34.58	92.91	−0.333
AtSUS2	*AT5G49190*	807	92,064.11	5.7	38.56	91.95	−0.241
AtSUS3	*AT4G02280*	809	92,002.08	5.85	42.06	88.31	−0.276
AtSUS4	*AT3G43190*	808	93,002.67	6.12	33.51	92.67	−0.3
AtSUS5	*AT5G37180*	837	95,034.46	6.13	36.23	86.2	−0.323
AtSUS6	*AT1G73370*	942	106,875.57	8.13	36.91	84.56	−0.391
BrSUS1-1	*BraA10g020200*	805	92,207.77	5.8	35.38	93.48	−0.299
BrSUS1-2	*BraA03g010160*	614	70,677.95	5.67	36.8	89.87	−0.356
BrSUS2	*BraA06g037460*	807	91,991.01	5.94	37.93	90.84	−0.237
BrSUS3	*BraA09g001900*	811	92,336.53	5.83	41.26	88.82	−0.274
BrSUS5	*BraA05g015600*	846	96,212.9	6.18	35.31	87.48	−0.339
BrSUS6-1	*BraA07g030030*	735	82,818.13	6.1	35.6	87.28	−0.247
BrSUS6-2	*BraA07g038750*	943	107,044.64	6.42	38.5	83.01	−0.37
BniSUS1-1	*BniB02g044340*	805	92,287.93	5.9	34.72	94.32	−0.3
BniSUS1-2	*BniB08g010690*	786	90,089.49	5.98	36.06	94.76	−0.339
BniSUS2	*BniB02g075900*	787	89,597.48	5.87	38.01	92.03	−0.197
BniSUS3	*BniB08g048340*	811	92,487.71	5.93	39.96	89.06	−0.295
BniSUS6-1	*BniB03g041770*	738	83,849.41	8.45	32.33	83.74	−0.372
BniSUS6-2	*BniB03g056480*	938	106,504.99	6.62	38.38	82	−0.385
BoSUS1-1	*BolC03g010920*	806	92,470.04	5.67	34.45	94.09	−0.308
BoSUS1-2	*BolC09g051640*	805	92,300.94	5.77	36.34	94.09	−0.296
BoSUS2	*BolC07g038200*	893	102,196.89	6.54	39.64	89.73	−0.238
BoSUS3	*BolC09g001700*	811	92,412.63	5.85	41.52	88.69	−0.281
BoSUS5	*BolC06g017370*	837	95,139.67	6.18	34.9	87.84	−0.334
BoSUS6-1	*BolC06g032260*	940	106,523.07	7.2	34.42	82.66	−0.382
BoSUS6-2	*BolC06g044870*	934	106,248.74	6.24	36.96	83.39	−0.363
BjSUS1-1	*BjuA047153*	805	92,250.8	5.8	35.45	93.35	−0.306
BjSUS1-2	*BjuB015313*	806	92,402.1	5.9	33.99	94.34	−0.304
BjSUS1-3	*BjuA009339*	806	92,478.08	5.67	36.08	94.09	−0.31
BjSUS1-4	*BjuO008945*	774	88,543.68	5.83	35.08	95.7	−0.276
BjSUS2-1	*BjuA023848*	807	91,949.91	5.89	38	90.84	−0.233
BjSUS2-2	*BjuB037515*	807	92,020.15	5.86	38.01	91.69	−0.213
BjSUS3-1	*BjuO006586*	811	92,507.72	5.93	40.64	88.1	−0.304
BjSUS3-2	*BjuA036504*	802	91,391.52	5.99	40.77	88.97	−0.27
BjSUS5-1	*BjuB022852*	755	85,420.14	5.82	35.13	86.93	−0.319
BjSUS5-2	*BjuA018844*	793	90,116.11	6.2	35.96	89.02	−0.333
BjSUS6-1	*BjuB030220*	905	102,929.82	6.21	37.31	82.2	−0.372
BjSUS6-2	*BjuB030962*	904	102,470.35	6.36	32.66	83.47	−0.359
BjSUS6-3	*BjuB047347*	904	102,481.14	6.17	32.42	82.61	−0.377
BjSUS6-4	*BjuA043452*	854	97,094	6	38.62	82.54	−0.344
BcSUS1-1	*BcaB08g36532*	833	95,392.62	5.95	35.68	94.43	−0.269
BcSUS1-2	*BcaB01g04767*	796	91,365.9	5.95	34.01	93.58	−0.318
BcSUS1-3	*BcaC04g19471*	795	91,173.69	5.89	36.5	91.72	−0.335
BcSUS2-1	*BcaC06g30710*	807	92,051.17	5.9	37.43	91.2	−0.228
BcSUS2-2	*BcaNung02586*	831	94,999.44	6.09	38.15	89.75	−0.252
BcSUS3-1	*BcaC04g24052*	811	92,427.64	5.88	42.3	88.69	−0.282
BcSUS3-2	*BcaB01g02060*	811	92,507.72	5.93	40.64	88.1	−0.304
BcSUS5-1	*BcaB02g08033*	837	95,295.82	6.45	37.3	87.37	−0.349
BcSUS5-2	*BcaC08g44340*	837	95,186.77	6.15	35.24	88.3	−0.328
BcSUS6-1	*BcaC08g46761*	934	106,194.82	6.5	37.26	83.39	−0.359
BcSUS6-2	*BcaB03g15055*	938	106,504	6.77	37.61	81.8	−0.393
BcSUS6-3	*BcaB03g15709*	929	105,215.74	7.48	35.52	84.28	−0.367
BcSUS6-4	*BcaC08g45643*	917	104,093.38	7.98	34.47	82.6	−0.398
BcSUS6-5	*BcaB03g15710*	934	105,917.91	8.12	34.1	86.31	−0.325
BcSUS6-6	*BcaC08g45646*	904	102,385.7	7.5	36.43	85.75	−0.362
BcSUS6-7	*BcaC08g45645*	889	100,722.42	6.96	35.6	84.56	−0.37
BcSUS6-8	*BcaB03g15711*	476	53,975.48	5.7	33.63	80.5	−0.314
BnSUS1-1	*BnaA10G0171200ZS*	839	96,123.24	5.9	36.08	91.19	−0.312
BnSUS1-2	*BnaC09G0457100ZS*	805	92,300.94	5.77	36.34	94.09	−0.296
BnSUS1-3	*BnaA03G0090400ZS*	806	92,478.12	5.71	35.43	94.09	−0.311
BnSUS1-4	*BnaC03G0102700ZS*	1363	156,032.04	7.26	38.75	91.38	−0.308
BnSUS2-1	*BnaC07G0334600ZS*	809	92,376.56	6.09	38.48	91.33	−0.223
BnSUS2-2	*BnaA06G0358400ZS*	743	84,612.73	6.03	39.38	92.1	−0.188
BnSUS3-1	*BnaA09G0024200ZS*	811	92,376.58	5.88	40.63	88.82	−0.279
BnSUS3-2	*BnaC09G0008900ZS*	811	92,383.63	5.93	41.91	89.05	−0.277
BnSUS5-1	*BnaA05G0143000ZS*	846	96,212.9	6.18	35.31	87.48	−0.339
BnSUS5-2	*BnaC06G0154000ZS*	846	96,236.97	6.1	35.69	88.29	−0.331
BnSUS6-1	*BnaA07G0255900ZS*	940	106,343.75	6.77	33.08	83.81	−0.379
BnSUS6-2	*BnaA07G0338200ZS*	968	109,966.11	6.47	38.76	83.88	−0.338
BnSUS6-3	*BnaC06G0283800ZS*	940	106,405.95	7.77	33.7	82.55	−0.386
BnSUS6-4	*BnaC06G0397800ZS*	907	103,109.05	6.32	37.42	81.37	−0.381

**Table 2:** Statistical summary of Ka/Ks ratios for *SUS* gene pairs across the U Triangle species.

Comparison Group	Gene Pairs Number	Mean Ka/Ks	Median Ka/Ks	Range (Min–Max)	% < 0.5	% < 1.0
AA-AABB (*Br* vs. *Bj*)	18	0.1075	0.1024	0.0257–0.2888	100%	100%
BB-AABB (*Bni* vs. *Bj*)	16	0.1300	0.1308	0.0713–0.1954	100%	100%
AA-AACC (*Br* vs. *Bn*)	15	0.1129	0.0788	0.0000–0.3932	100%	100%
CC-AACC (*Bol* vs. *Bn*)	19	0.1015	0.0935	0.0000–0.2914	100%	100%
BB-BBCC (*Bni* vs. *Bc*)	15	0.1306	0.1367	0.0633–0.1963	100%	100%
CC-BBCC (*Bol* vs. *Bc*)	20	0.1541	0.1127	0.0440–0.4938	100%	100%
Total/Overall	103	0.1218	0.1109			

## Data Availability

All additional datasets supporting the findings of this study are included within the article and Supplementary Materials.

## Supplementary Materials

The following supporting information can be downloaded at https://www.mdpi.com/article/10.3390/plants15081224/s1: Figure S1: Quantitative profiling and functional categorization of *cis*-acting regulatory elements in the *SUS* promoters of six *Brassica* species. Figure S2: Chromosomal distribution and localization of *SUS* gene families in *A. thaliana* and six *Brassica* species. Figure S3: Molecular identification and expression analysis of *BnSUS3-2* in transgenic *Arabidopsis*. Table S1: Ka/Ks analysis of syntenic *SUS* gene pairs across six *Brassica* species. Table S2: Primer names and sequences used in this study.
