# Supplementary material for: Genome-Wide Identification and Evolutionary Analysis of Sucrose Synthase (SUS) Gene Family in U’s Triangle Brassica Species
Source: Plants (Basel). 2026 Apr 16;15(8):1224. doi: 10.3390/plants15081224 (PMC13120658; doi:10.3390/plants15081224)
Supplement: Supplementary file 1 [file plants-15-01224-s001.zip › plants-4214639-supplementary.pdf]

Supplementary Materials

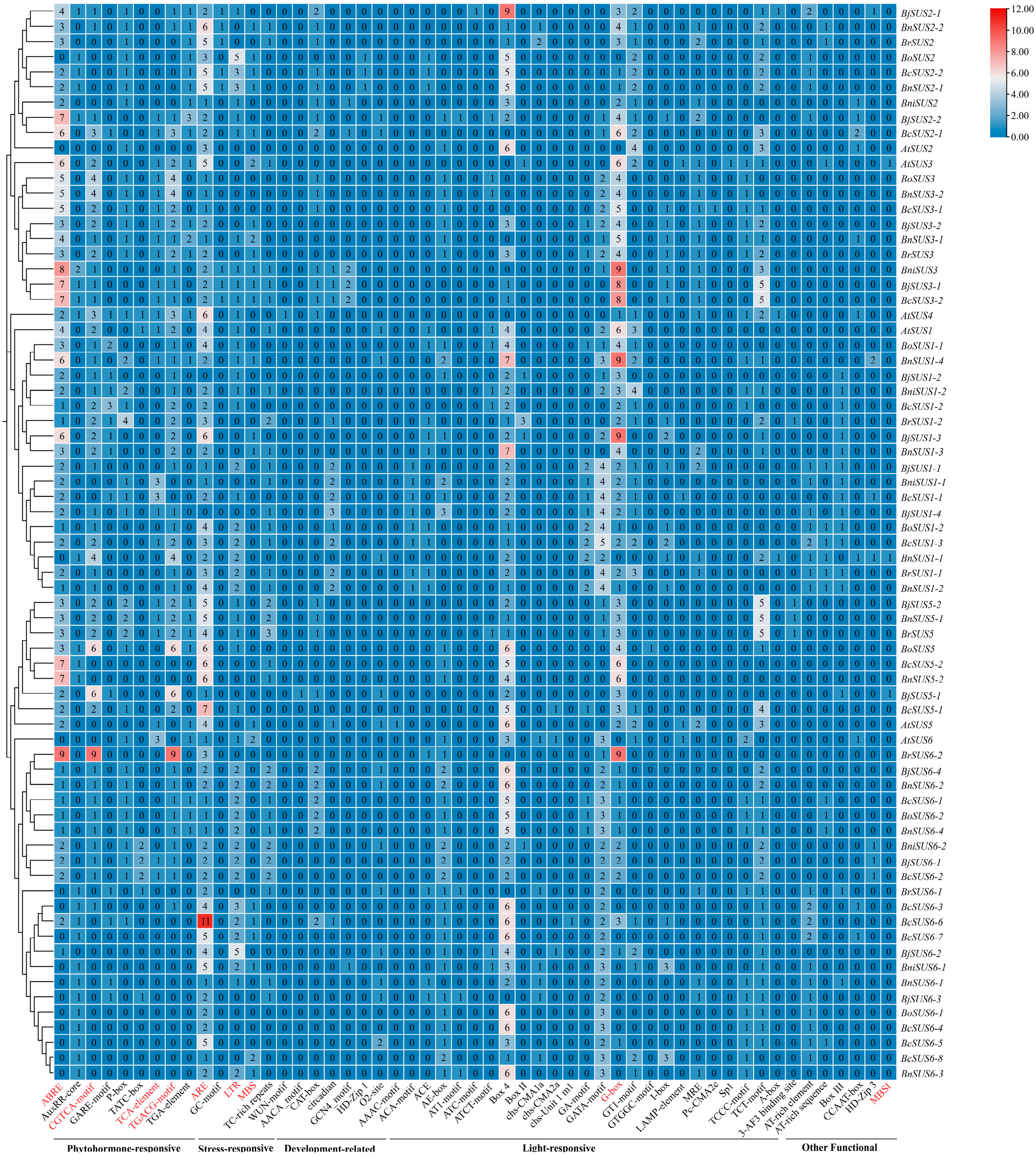

Figure S1. Quantitative profiling and functional categorization of *cis*-acting regulatory elements in the *SUS* promoters of six *Brassica* species. The heat map (or bar chart) illustrates the abundance of specific *cis*-elements identified within the 2 kb upstream regions from the translation start site (TSS).

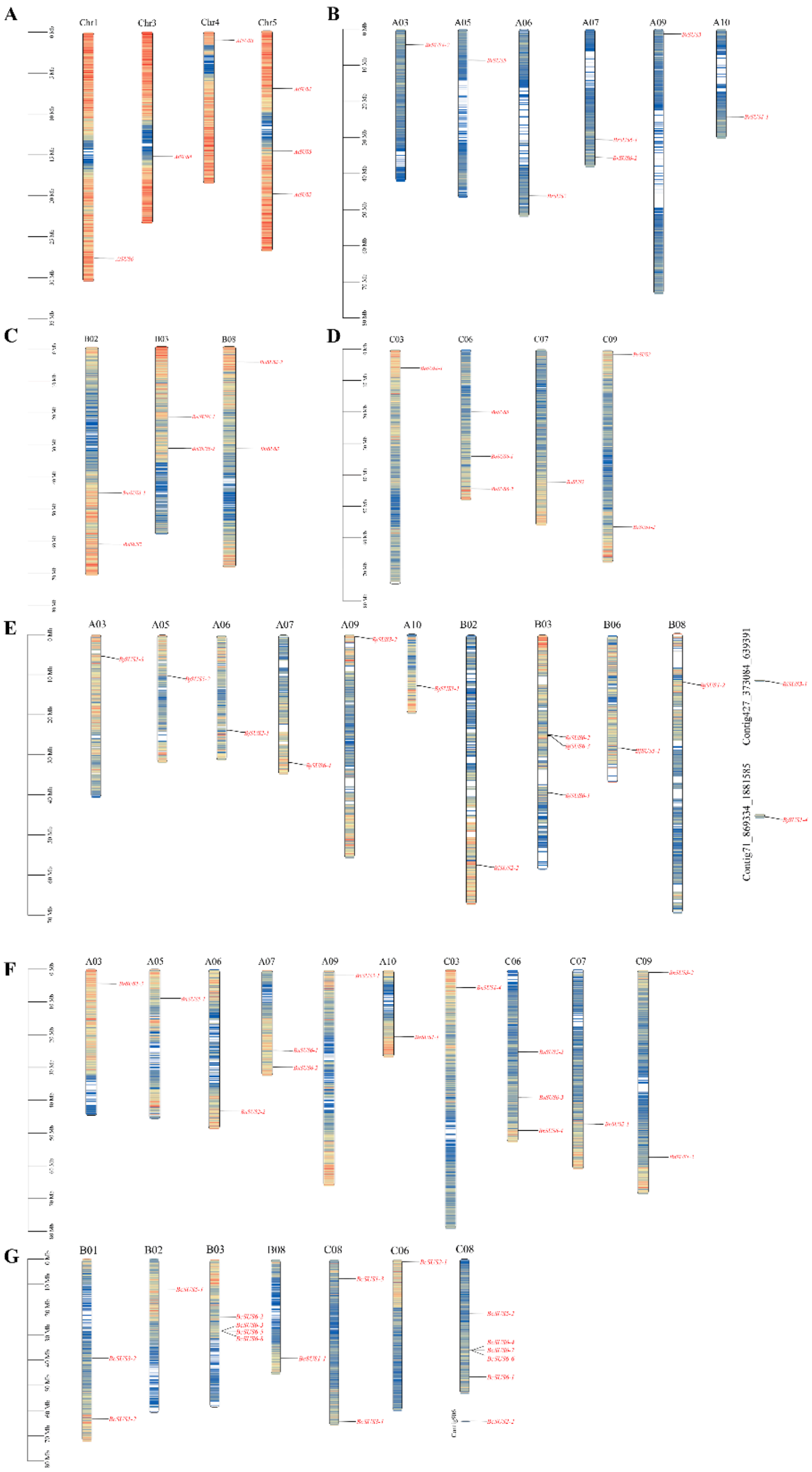

Figure S2. Chromosomal distribution and localization of *SUS* gene families in *A. thaliana* and six *Brassica* species. The physical positions of *SUS* genes were mapped onto the respective chromosomes based on genome annotation files: **(A)** *B. rapa* (AA); **(B)** *B. nigra* (BB); **(C)** *B. oleracea* (CC); **(D)** *B. juncea* (AABB); **(E)** *B. napus* (AACC); and **(F)** *B. carinata* (BBCC). Chromosome numbers and scales (in Mb) are indicated for each species to show the relative gene density and genomic distribution.

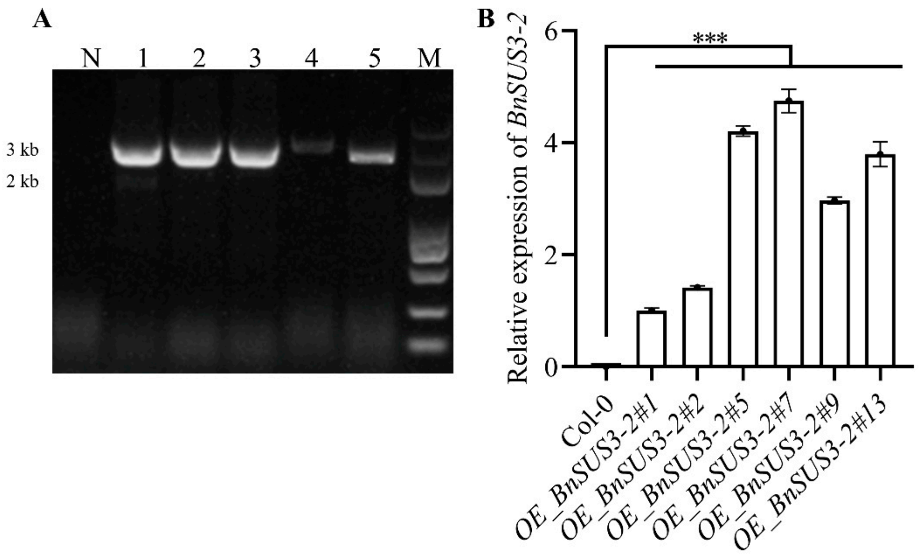

Figure S3. Molecular identification and expression analysis of *BnSUS3-2* in transgenic *Arabidopsis*. **(A)** Genomic DNA PCR identification of the *BnSUS3-2* overexpressing lines. The "N" represents the negative control (template-free control). **(B)** Transcript levels of *BnSUS3-2* in different transgenic lines determined by qRT-PCR. The relative expression level was calculated using the $\Delta\Delta^{Ct}$ value method, with the expression value of transgenic line 1 (#1) used as the baseline control. Data are presented as the means SD of three biological replicates.

Table S1. Ka/Ks analysis of syntenic *SUS* gene pairs across six *Brassica* species

Paralogous gene pair	Ka	Ks	Ka/Ks
BrSUS1-1/BjSUS1-3	0.0149	0.3223	0.0463
BrSUS1-1/BjSUS1-1	0.0022	0.0837	0.0257
BrSUS1-1/BjSUS1-2	0.0177	0.2588	0.0683
BrSUS1-1/BjSUS1-4	0.0079	0.1476	0.0534
BrSUS2/BjSUS2-1	0.0019	0.0501	0.0378
BrSUS2/BjSUS2-2	0.0111	0.1932	0.0577
BrSUS3/BjSUS3-2	0.0011	0.0293	0.0370
BrSUS3/BjSUS3-1	0.0159	0.2233	0.0714
BrSUS5/BjSUS5-2	0.0049	0.0486	0.1004
BrSUS5/BjSUS5-1	0.0092	0.0953	0.0964
BrSUS6-1/BjSUS6-4	0.0486	0.3586	0.1355
BrSUS6-1/BjSUS6-3	0.0232	0.1111	0.2084
BrSUS6-1/BjSUS6-1	0.0458	0.3247	0.1411
BrSUS6-1/BjSUS6-2	0.0331	0.2446	0.1352
BrSUS6-2/BjSUS6-4	0.0010	0.0035	0.2888
BrSUS6-2/BjSUS6-1	0.0229	0.2115	0.1082
BrSUS6-2/BjSUS6-2	0.0349	0.2823	0.1235
BrSUS6-2/BjSUS6-3	0.0337	0.3230	0.1043
BjSUS1-1/BniSUS1-2	0.0393	0.2783	0.1412
BjSUS1-2/BniSUS1-2	0.0266	0.1375	0.1931
BjSUS1-3/BniSUS1-2	0.0309	0.2322	0.1330
BjSUS1-4/BniSUS1-2	0.0368	0.2732	0.1347
BjSUS2-1/BniSUS2	0.0184	0.2168	0.0848
BjSUS2-2/BniSUS2	0.0077	0.0786	0.0978
BjSUS3-1/BniSUS3	0.0016	0.0090	0.1791
BjSUS3-2/BniSUS3	0.0156	0.2183	0.0713
BjSUS6-1/BniSUS6-2	0.0033	0.0442	0.0752
BjSUS6-1/BniSUS6-1	0.0364	0.2845	0.1280
BjSUS6-2/BniSUS6-2	0.0429	0.2784	0.1542
BjSUS6-2/BniSUS6-1	0.0141	0.0721	0.1954
BjSUS6-3/BniSUS6-1	0.0204	0.1587	0.1285
BjSUS6-3/BniSUS6-2	0.0400	0.3202	0.1248
BjSUS6-4/BniSUS6-2	0.0212	0.2139	0.0993
BjSUS6-4/BniSUS6-1	0.0309	0.2853	0.1083
BrSUS1-1/BnSUS1-3	0.0161	0.3136	0.0514
BrSUS1-1/BnSUS1-1	0.0016	0.0837	0.0193

BrSUS1-1/BnSUS1-2	0.0054	0.1082	0.0499
BrSUS2/BnSUS2-1	0.0076	0.1236	0.0618
BrSUS3/BnSUS3-1	0.0011	0.0290	0.0369
BrSUS5/BnSUS5-1	0.0000	0.0000	NaN
BrSUS5/BnSUS5-2	0.0061	0.0527	0.1163
BrSUS6-1/BnSUS6-1	0.0226	0.0574	0.3932
BrSUS6-1/BnSUS6-2	0.0459	0.3522	0.1302
BrSUS6-1/BnSUS6-3	0.0275	0.1444	0.1903
BrSUS6-1/BnSUS6-4	0.0489	0.3449	0.1419
BrSUS6-2/BnSUS6-2	0.0023	0.0289	0.0788
BrSUS6-2/BnSUS6-1	0.0333	0.3178	0.1048
BrSUS6-2/BnSUS6-4	0.0086	0.1330	0.0644
BrSUS6-2/BnSUS6-3	0.0323	0.2813	0.1149
BoSUS1-1/BnSUS1-3	0.0038	0.1335	0.0282
BoSUS1-1/BnSUS1-1	0.0152	0.2961	0.0514
BoSUS1-1/BnSUS1-4	0.0016	0.0110	0.1485
BoSUS1-1/BnSUS1-2	0.0166	0.3188	0.0520
BoSUS1-2/BnSUS1-3	0.0161	0.3213	0.0502
BoSUS1-2/BnSUS1-1	0.0038	0.1123	0.0336
BoSUS1-2/BnSUS1-2	0.0000	0.0036	0.0000
BoSUS2/BnSUS2-1	0.0027	0.0144	0.1886
BoSUS3/BnSUS3-1	0.0064	0.0689	0.0935
BoSUS5/BnSUS5-1	0.0057	0.0512	0.1109
BoSUS5/BnSUS5-2	0.0015	0.0053	0.2914
BoSUS6-1/BnSUS6-1	0.0097	0.0878	0.1104
BoSUS6-1/BnSUS6-2	0.0335	0.2801	0.1196
BoSUS6-1/BnSUS6-3	0.0028	0.0095	0.2909
BoSUS6-1/BnSUS6-4	0.0346	0.2945	0.1174
BoSUS6-2/BnSUS6-2	0.0083	0.1249	0.0666
BoSUS6-2/BnSUS6-1	0.0348	0.3029	0.1149
BoSUS6-2/BnSUS6-4	0.0000	0.0000	NaN
BoSUS6-2/BnSUS6-3	0.0338	0.2897	0.1167
BniSUS1-2/BcSUS1-2	0.0200	0.1034	0.1931
BniSUS1-2/BcSUS1-1	0.0370	0.2782	0.1329
BniSUS1-2/BcSUS1-3	0.0572	0.2913	0.1963
BniSUS2/BcSUS2-1	0.0080	0.0676	0.1179
BniSUS2/BcSUS2-2	0.0158	0.1971	0.0803
BniSUS3/BcSUS3-2	0.0016	0.0090	0.1791
BniSUS3/BcSUS3-1	0.0146	0.2300	0.0633

BniSUS6-1/BcSUS6-3	0.0065	0.0634	0.1027
BniSUS6-1/BcSUS6-2	0.0368	0.2961	0.1245
BniSUS6-1/BcSUS6-4	0.0249	0.1795	0.1387
BniSUS6-1/BcSUS6-1	0.0407	0.2977	0.1367
BniSUS6-2/BcSUS6-2	0.0028	0.0425	0.0646
BniSUS6-2/BcSUS6-3	0.0402	0.2750	0.1463
BniSUS6-2/BcSUS6-1	0.0249	0.2431	0.1025
BniSUS6-2/BcSUS6-4	0.0451	0.2799	0.1612
BoSUS1-1/BcSUS1-2	0.0103	0.2029	0.0507
BoSUS1-1/BcSUS1-1	0.0144	0.3273	0.0440
BoSUS1-1/BcSUS1-3	0.0328	0.3548	0.0924
BoSUS1-2/BcSUS1-2	0.0211	0.2746	0.0770
BoSUS1-2/BcSUS1-1	0.0081	0.1776	0.0457
BoSUS1-2/BcSUS1-3	0.0173	0.0350	0.4938
BoSUS2/BcSUS2-1	0.0081	0.1811	0.0449
BoSUS2/BcSUS2-2	0.0010	0.0086	0.1212
BoSUS3/BcSUS3-2	0.0140	0.2224	0.0631
BoSUS3/BcSUS3-1	0.0021	0.0366	0.0585
BoSUS5/BcSUS5-1	0.0140	0.1036	0.1354
BoSUS5/BcSUS5-2	0.0015	0.0053	0.2914
BoSUS6-1/BcSUS6-3	0.0219	0.1815	0.1208
BoSUS6-1/BcSUS6-2	0.0399	0.2801	0.1425
BoSUS6-1/BcSUS6-4	0.0064	0.0138	0.4593
BoSUS6-1/BcSUS6-1	0.0326	0.2971	0.1098
BoSUS6-2/BcSUS6-2	0.0268	0.2321	0.1156
BoSUS6-2/BcSUS6-3	0.0442	0.2837	0.1559
BoSUS6-2/BcSUS6-1	0.0018	0.0211	0.0869
BoSUS6-2/BcSUS6-4	0.0379	0.2991	0.1266

Table S2. Primer names and sequences used in this study

Primer name	Primer sequence
F-25S-RT	GATTTCTGCCCAGTGCTCTGAA
R-25S-RT	TCTGCCAAGCCCGTTCCCTT
F-BnSUS1-1-RT	CCACCATTCACTGAAACCCCT
R-BnSUS1-1-RT	TCGTTCAAACGCTCACGTTG
F-BnSUS1-2-RT	TCCAGGGTTGAAGGCAAAGG
R-BnSUS1-2-RT	GCTATCCATGGTGGCAACAC
F-BnSUS1-3-RT	TGCACGGCAAATCGGGA
R-BnSUS1-3-RT	TCCTCAATCCTCTGAAGCCC
F-BnSUS1-4-RT	TGGTTGGAATGGTAGTGGAGTC
R-BnSUS1-4-RT	CAGCAACACGTTTCAGTCCG
F-BnSUS2-1-RT	ACGCTGGCTGGTGTCTATTC
R-BnSUS2-1-RT	AGGCGAAGAAATTTAAAGAAGAAGA
F-BnSUS2-2-RT	TCACTGCGGATCTAATTGCCA
R-BnSUS2-2-RT	TGATTCCGTGAACAACTCTGT
F-BnSUS3-1-RT	TTGAGCGATAGGTCGAAGCC
R-BnSUS3-1-RT	TTACGACCAGGTTCACCAGC
F-BnSUS3-2-RT	TCGGATCCAAAGCATATCTAGG
R-BnSUS3-2-RT	GCACTCGTCCCACTGTATCT
F-BnSUS5-1-RT	CAGCTCAAACTGATCGGACAC
R-BnSUS5-1-RT	CCATCCATGATAATCTCGGCAGGA
F-BnSUS5-2-RT	TTGAGAAGGGCAGAAGGCTG
R-BnSUS5-2-RT	TTCCTCGTACCGCAAATGCT
F-BnSUS6-1-RT	AAGGCAGGAGAAGGAGAGGT
R-BnSUS6-1-RT	GAGGCAAACCACTACGGACA
F-BnSUS6-2-RT	CTTCACAAGTTATCTCACTCTGGT
R-BnSUS6-2-RT	ATAAGGCGTGTGTTTGGGGT
F-BnSUS6-3-RT	GGTTTCCCGGTTCGATGTCT
R-BnSUS6-3-RT	CCAACGATGAGGTCAGGCTT
F-BnSUS6-4-RT	AGGCCAGCCTCAGATTCTTG
R-BnSUS6-4-RT	CGTGAAACCCATTGACGGAG
AtActin-F	GCAGACCGTATGAGCAAAG
AtActin-R	GGAAGCAAGGATAGAACCAC
1300-SUS3-2-F	CGAGCTTTCGCGAGCTCGGTACCATGTCAAACCCTAGACTCACT
1300-SUS3-2-R	CTCGCCCTTGCTCACCATGGATCCTTCATCGTCGCTTAAAGGTAC
